# Allometry indicates giant eyes of giant squid are not exceptional

**DOI:** 10.1186/1471-2148-13-45

**Published:** 2013-02-18

**Authors:** Lars Schmitz, Ryosuke Motani, Christopher E Oufiero, Christopher H Martin, Matthew D McGee, Ashlee R Gamarra, Johanna J Lee, Peter C Wainwright

**Affiliations:** 1 Department of Evolution and Ecology, University of California, Davis, CA 95616, USA; 2 Department of Geology, University of California, Davis, CA 95616, USA; 3 W.M. Keck Science Department, Claremont McKenna College, Pitzer College, and Scripps College, Claremont, CA 91711, USA; 4 Department of Biological Sciences, Towson University, Towson, MD 21252, USA

## Abstract

**Background:**

The eyes of giant and colossal squid are among the largest eyes in the history of life. It was recently proposed that sperm whale predation is the main driver of eye size evolution in giant squid, on the basis of an optical model that suggested optimal performance in detecting large luminous visual targets such as whales in the deep sea. However, it is poorly understood how the eye size of giant and colossal squid compares to that of other aquatic organisms when scaling effects are considered.

**Results:**

We performed a large-scale comparative study that included 87 squid species and 237 species of acanthomorph fish. While squid have larger eyes than most acanthomorphs, a comparison of relative eye size among squid suggests that giant and colossal squid do not have unusually large eyes. After revising constants used in a previous model we found that large eyes perform equally well in detecting point targets and large luminous targets in the deep sea.

**Conclusions:**

The eyes of giant and colossal squid do not appear exceptionally large when allometric effects are considered. It is probable that the giant eyes of giant squid result from a phylogenetically conserved developmental pattern manifested in very large animals. Whatever the cause of large eyes, they appear to have several advantages for vision in the reduced light of the deep mesopelagic zone.

## Background

The eyes of giant squid (*Architeuthis* spp.) and colossal squid (*Mesonychoteuthis hamiltoni*) are the largest of all living organisms [1]. In adult individuals the eyes can be as large as 27 cm, about three times the largest diameter of swordfishes (*Xiphias gladius*) with approximately the same body size, considered to have the largest eyes among living teleost fish [1]. Direct observations of the biology of giant and colossal squid are extremely rare and thus the function and performance of their large eyes is not well understood. As a result, theoretical models offer a particularly important perspective on their visual performance [2], on which basis one can develop evolutionary hypotheses about eye size evolution. For example, it has been suggested recently that the giant eyes of giant squid are especially well-suited to detect large luminous objects in the dim-light environment of the deeper part of the mesopelagic realm, at water depths of 600–1000 m [1]. According to the general conclusions of this model, larger eyes perform better in detecting large, black objects and bright bioluminescent light flashes yet the detection of form illumination provides the largest visual range. Sperm whale detection by squid may involve form illumination because whales are thought to induce bioluminescence in particular in areas with high zooplankton density, suggesting the possibility that whale detection was the driver of extreme eye size evolution in giant and colossal squid [1]. However, even though the eyes of these squid may be much larger than the eyes of similarly-sized fish as indicated by the comparison of giant squid to swordfish, they may not be out of proportion compared to other squid [2]. Here we account for body size while asking whether giant and colossal squid have unusually large eyes among a broad sample of squid species, and whether squid eyes are larger than a broad interspecific sample of acanthomorph fishes that represent a clade with independently evolved camera-eyes functionally similar to squid eyes. In addition, we revisit the optical benefits of large eye size in the deep mesopelagic sea by revising the constants used in a visual performance model [1].

## Methods

We investigated two different regression problems: (1) relative eye size (i.e., eye size for a given body size) among squid; (2) relative eye size across squid and acanthomorph fish. Both problems deal with eye size for a given body size, or, in other words, the relation between eye size and body size. The preferred method for this question is major axis (MA) or standardized major axis (SMA) line-fitting techniques because we attempted to identify the slope of the line that best characterizes the relation between body size and eye size [3]. In addition we decided to explore in how far results may be influenced by choice of method and present ordinary least square regression (OLS) results along with the SMA results. OLS is useful if one was interested in calculating the expected eye size for a specific body size, yet is known to often underestimate the true slope, especially when the correlation between the two variables is low [4]. We did not correct for the phylogenetic covariance among species because of the unavailability of complete species level phylogenies for squid and acanthomorph fish. We performed all calculations in R 2.14.2 [5], including the robust SMA line estimates with Huber’s *M*-estimators [6,7]. All squid measurements were taken from either preserved specimens stored in museum collections or the primary literature; no live animals were used for this part of the project. In order to compare squid eye size to that of acanthomorph fish, we measured fish specimens in museum collections. Finally, we measured external and full eye diameter in teleost fish in unfixed specimens. All research was carried out in accordance with the UC Davis animal use and care protocol.

In order to address relative eye size within squid, we measured eye diameter and dorsal mantle length in 58 species and supplemented this dataset with published data on 29 species (Additional files 1 and 2). For eye diameter, we measured the largest diameter of the exposed portion of the eye. If integument covered large parts of the eye we only measured eye diameter when the eye was clearly discernible as a prominent, well-defined bulge underneath the integument. Dorsal mantle length is a commonly used body-size proxy for squid [8]. Using mantle length as the size proxy also renders it possible to include many published data for which reliable body mass information is not available.

In order to test whether *Architeuthis* and *Mesonychoteuthis* (the giant and colossal squid) have unusually large eyes compared to other squids we first calculated the SMA line and evaluated where in relation to this line the giant and colossal squid fall. We did not include *Architeuthis* and *Mesonychoteuthis* in this part of the analysis in order to avoid a bias in the regression analysis. Many of the giant squid individuals are larger than the squid species used in the calculation of the SMA line, and we assumed the scaling trend continues for larger body sizes. In order to test whether choice of regression method influenced results, we also performed OLS regression. As mentioned above, OLS can be useful for making a prediction of the dependent variable on the basis of a specific independent variable, but often underestimates the regression slope, in particular for low correlations [4]. We calculated the 95% prediction belts of the OLS regression of eye and body size for all squid except for giant and colossal squid. The prediction belts were calculated on the basis of a single observation of body size (n=1) and describe the range around the expected mean of eye diameter with a 95% probability to contain the true mean. In other words, the prediction range describes what eye size is expected for a given body size. We then examined where giant and colossal squid plot in the distribution of all other squid. Unexpectedly large eyes for a given body size would be indicated if they plotted above the 95% prediction belt. We assessed the robustness of the prediction interval by resampling (with replacement) and also estimated a *P*-value with a parametric bootstrap. We emphasize the importance of calculating prediction intervals in order to formally address the question whether the eyes of giant and colossal squid are unusual when using OLS regression. A regression line itself contains uncertainty and thus it is important to take this uncertainty into account when evaluating whether a trait is unusual, i.e., plotting outside an expected distribution. Simple observations of samples that plot above and below the regression line are not informative, because about half the samples lie above the regression line by default.

The second regression problem deals with the comparison of relative eye size in squid and acanthomorph fish. This is an unusual comparison because squid (cephalopods) and fish (vertebrates) are phylogenetically distant. However, the comparison is possible because both squid and acanthomorphs are highly visual, aquatic animals with camera-type eyes and similar properties of their refractive system [9]. A challenging problem is to identify a common body size proxy in these distantly related clades. We chose body mass as the size proxy, because mass should be a less biased proxy than any linear measurements of body size. As we were interested in the scaling of a linear variable (eye diameter), and to facilitate comparisons to the squid-only patterns, we used the cuberoot of body mass as the independent variable. We collected eye diameter and body mass measurements with calipers (0.1 mm accuracy) and a digital scale (0.1 g accuracy), respectively.

For fish, we sampled across a very large range of species within Acanthomorpha (spiny-rayed fishes), a clade that includes a large part of the biodiversity of teleost fishes (>16,000 species) and is known for widely varying relative eye size [10]. We measured the largest exposed eye diameter and body mass in fixed specimens for a total of 237 acanthomorph species representing 237 families, a phylogenetically and ecologically very broad sample (Additional file 3). For squid, we measured eye diameter (see above) and body mass in fixed specimens for a total of 58 species. It should be noted that the exposed eye diameter may be slightly smaller than the total equatorial diameter of the eye, and some noise may be introduced to the analysis by interspecific differences in the relation between exposed/total eye diameters. We tested this by comparing the exterior and total eye diameters of 273 species of unfixed teleost fish and found a near 1-to-1 relation (isometric slope, intercept not different from 0; for data please see Additional file 4). We do not have data on squid. This would only be a problem if there is a systematic change in the proportion of the eye that is covered as squid grow, because it would affect the slope of the regression which is critical in the evaluation of whether the big squid are exceptional. After checking the fish data such a bias is unlikely but it cannot be fully excluded. Another possible source of error is the type of specimen preservation. We minimized this error by analyzing specimens with the same preservation type. Both fish and squid datasets contain body mass of formalin-fixed and ethanol-preserved specimens, and thus there should be no systematic bias in comparing relative eye size between fish and squid. We did not include giant squid in this part of the analysis because their body mass was measured in unfixed specimens (e.g., [11]). A mix of unfixed and ethanol-preserved specimens may introduce a bias in comparing relative eye size because of differential weight loss depending on preservation of specimens [12]. We compared relative eye size in squid and fish with an ANCOVA.

**Figure 1:**
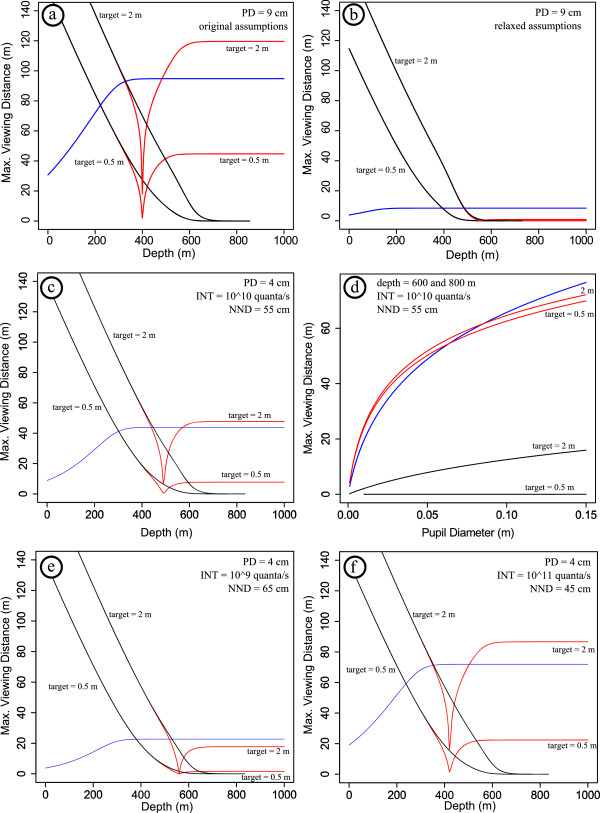
**Visual performance in the deep sea.** Results of the revised visual performance model are expressed as maximum viewing distance for three different types of targets: point sources against black background (blue line), extended luminous targets (red lines, 0.5 and 2 m target diameter), and dark extended sources (black lines, 0.5 and 2 m target diameter). (**a**) Visual performance model on the basis of original model assumptions from ref. 1 (**b**) Visual performance model on the basis of relaxed model assumptions. Please see text for further explanation. (**c**) Visual performance model on the basis of revised estimates of eye size (pupil diameter, PD = 4 cm), bioluminescent photon flux intensity (INT = 10^10^ quanta/s), and mesopelagic zooplankton density (nearest neighbor distance, NND = 55 cm). These parameter estimates are reasonable values for large parts of the deep mesopelagic realm. (**d**) Maximum viewing distance as a function of pupil size for the deep mesopelagic realm (600 and 800 m depth), demonstrating similar performance of point-light (against black background) and extended luminous target detection for a large range of eye sizes. (**e**) and (**f**) illustrate the effects of varying the parameter estimates within the ranges that may occasionally be encountered by giant squid in the deep mesopelagic settings. In areas with very high density of plankton with high bioluminescent photon flux large luminous sources can be detected about 10 m further away than point light sources when observed by an eye with 4 cm pupil diameter. In areas with very low density of plankton with low photon flux point light sources can be detected over a slightly larger distance. Again, most reasonable estimates of viewing distances in the deep mesopelagic realm are shown in (**c**).

In order to assess the optical performance of large eyes, we implemented and numerically solved the equations of the visual performance model of [1] in R 2.14.2 [5]. We did not modify the model itself, and confirmed that our implementation accurately reproduced the results [1] when the original constant values were used (Figure 1a). We revised the original parameter estimates for the model [1] following a review of the literature, focusing in particular on bioluminescent photon flux intensity and mesopelagic zooplankton density.

## Results

### Eye size among squid

**Figure 2:**
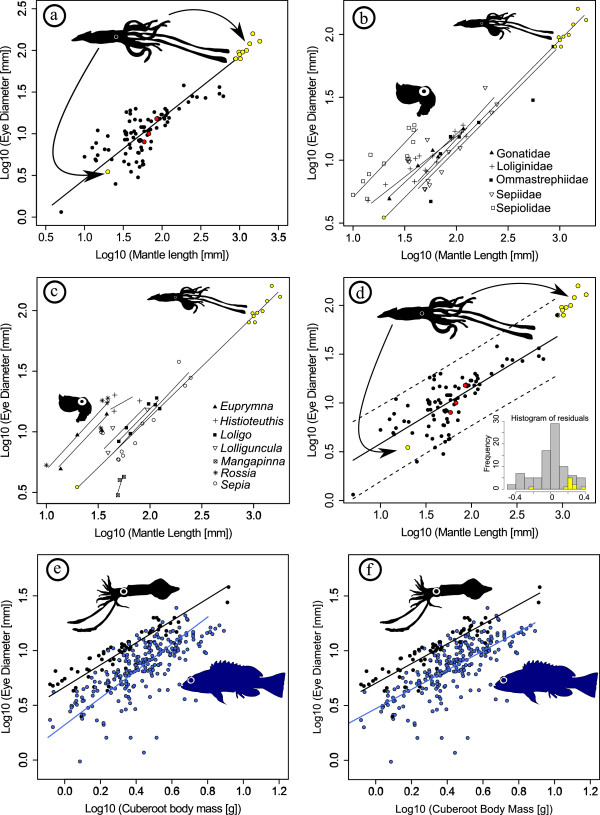
**Relative eye size in squid and fish.** (**a**) The plot of log10-transformed eye diameter against log10-transformed mantle length suggests that giant squid do not have unusually large eyes for their body size. All sampled individuals of giant squid (yellow) and colossal squid (red) fall directly on the projected SMA line calculated from averages of 85 squid species. (**b**) and (**c**) In particular sepiolids (e.g., *Rossia* and *Euprymna*) tend to have large eyes for given body size, but also Gonatidae and Loligonidae slightly exceed the relative eye size of giant squid. The fitted lines represent SMA lines. (**d**) The same conclusion can be drawn from an OLS regression, even though this method is known to underestimate the slope. All giant squid and colossal squid fall within the projected 95% prediction belts. The plot or residuals from the OLS regression line demonstrates that giant squid may have larger than average eyes, but not outside the distribution of other squid (**e**) The plot of log10-transformed eye diameter against log10-transformed cuberoot body mass shows that many squid (black) have larger relative eye size than acanthomorph fishes (blue). SMA lines suggest that eye size differences between squid and fish become smaller for larger body sizes. (**f**) OLS regression lines indicate that differences persist, with squid having on average about 1.7x the eye diameter of acanthomorph fish for a given body size.

Giant squid have very large body size and many of the sampled specimens are outside the size range of other sampled squid species, with the exception of the Humboldt squid *Dosidicus gigas*. If the trend of the SMA line (slope = 0.739; if the robust method [6] is chosen the slope increases to 0.742) continues for larger body sizes, then giant squid do not have unexpectedly large eye diameters for their mantle length (Figure 2a). All individuals with available data fall directly along the projected SMA line. The comparison to selected families and genera reveals the same pattern (Figures 2b, c). SMA lines for families and genera tend to have similar slopes as the SMA line for all *Architeuthis* individuals, and it is apparent that many families and genera have larger eyes for given mantle length than *Architeuthis*, in particular among the Sepiolidae with the genera *Euprymna* and *Rossia*. *Histioteuthis* has a much larger eye than *Architeuthis* as well, and also the loliginids *Loligo* and *Lolliguncula* and the Gonatidae have slightly larger eyes than the giant squid.

The same conclusion can be reached by OLS (slope = 0.566), despite the fact that SMA is better at estimating the slope. All giant and colossal squid individuals fall within the 95% prediction belt (Figure 2d). Even though *Architeuthis* may have larger eyes than the average squid according to OLS, it is within the distribution of all other sampled squid. For example, for a mantle length of 865 mm, corresponding to the size of the sampled Humboldt squid (*Dosidicus gigas*), the maximum eye diameter of the prediction range is 119.7 mm. The eye diameter of *Architeuthis* specimens at around this body size is approximately 80 mm. A plot of the residuals of the OLS regression, where giant squid fall within the distribution of all other squid, demonstrates that many squid species have relative eye sizes comparable to that of giant squid (Figure 2d). The resampling test indicates that the prediction interval is very robust: in 98.8% of replicates (n=10,000) *Architeuthis* (species average) plotted within the predicted range. Giant squid eyes were not significantly larger than other squid species (*P* = 0.979, parametric bootstrap resampling under the null hypothesis in which giant squid were sampled from the same normal linear model as estimated for other squid). An even stronger pattern emerges for *Mesonychoteuthis*. All sampled juvenile specimens plot very close to the regression line for all squid (Figure 2d). Resampling (n=10,000) did not yield a single case where *Mesonychoteuthis* (species average) plotted outside the prediction interval. The estimated *P*-value is >0.999.

### Eye size of squid and acanthomorph fish

When plotting log-transformed (base 10) eye diameter against log-transformed (base 10) cuberoot of body mass, it is apparent that most squid have very large eyes for a given body size, exceeding the eye size of most fish (Figures 2e, f). The SMA analysis (Figure 2e) indicates that eye size differences between acanthomorph fish and squid become smaller for larger body sizes, because the slopes were found to be different (1.231 and 1.015, respectively, *P*=0.012). Robust estimates of the SMA lines [6] are 1.125 for acanthomorph fish and 0.955 for squid. The results of an ANCOVA suggests that squid and fish have similar OLS slopes (0.904 and 0.879, respectively; Figure 2f) but different intercepts (0.702 and 0.471, respectively), i.e., squid have bigger eyes for a given body size (*P*<0.001). On average, squid have ~1.7 times the eye diameter of fish for a given body size.

## Discussion

The eyes of giant and colossal squid are among the largest eyes to evolve in the history of life. Only ichthyosaurs, fish-shaped marine reptiles of the Mesozoic, had comparable eye diameters [13]. It was previously concluded that the enormous eye sizes of giant and colossal squid are unusual for squid of this size [1], but this assessment was derived from a published regression line for an ontogenetic series of the myopsid squid *Loligo opalescens*[14], a phylogenetically distant relative of *Architeuthis*[15]. It is more reasonable to assess relative eye size with a large comparative dataset across squid species, as intra- and interspecific scaling patterns often differ.

On the basis of our regression analysis it is difficult to support the view that giant and colossal squid have unusually large eyes for their body size. Even though *Architeuthis* may have bigger eyes than average for squid, its eye size cannot be considered exceptional or unusual on the basis of the available data. All sampled individuals of *Architeuthis* and *Mesonychoteuthis* fall within the range of other squid. This implies that the giant eyes of giant and colossal squid could result from a phylogenetically conserved developmental pattern—in other words, their eye sizes are in the range predicted for any squid of that size. It is interesting that in particular the sepiolids have relatively large eyes, which in part may be a result of their proportionately shorter body appearance, indicating a phylogenetic influence. Sepiolids belong to a fairly basal clade within decapodiforms (15). However, even taxa more closely related to *Architeuthis* such as the Gonatidae have similar or larger eyes than the giant squid.

An important goal for future studies is to collect more data on eye and body size especially for very large individuals of giant and colossal squid, because these measurements are currently unavailable. For example, there is no published measurement of mantle length for the giant squid specimen with the reported eye diameter of 27 cm [1]. It will also be important to investigate whether the trend of the SMA line and linear regression holds true for very large body sizes. For the purposes of this study we assumed the linear regression is robust for large body sizes. If there is indication that the trend, i.e., the slope changes for large body sizes the question of the exceptionality of giant squid eyes needs to be re-evaluated.

In terms of absolute size, extant fish may not have eyes as large as the enormous eyes seen in squid. For example, eye diameters in large open ocean predators such as the swordfish (*Xiphias gladius*) do not exceed 9 cm [1] even though they forage in the deep mesopelagic realm [16] similar to *Architeuthis*[17]. Our results show that in addition to differences in absolute eye size there are differences in relative eye size (i.e., eye size for a given body size) between squid and fish. As we demonstrated in Figures 2c and d many squid have eye diameters unmatched by extant acanthomorph fishes for a given body mass. Squid have about 1.7 times larger eyes than fish of the same size. It is possible that squid are freed from structural constraints on eye size [18] because they do not have a rigid internal skeletal framework. But, potential variations in retinal structure between cephalopod and vertebrate camera eyes may be a more plausible explanation for these eye size differences. The unusually steep SMA slopes for both squids and acanthomorph fish can probably be explained by the presence of multiple sub-groups (e.g., nocturnal, diurnal) in each group that each have shallower slopes but different intercepts. By combining all sub-groups into one single group the resulting slope for the entire sample will be steeper.

Even if giant and colossal squid do not have unusually large eyes they may still benefit from a unique optical advantage, i.e., there is a best performance for a single optical function, according to work based on a model of visual performance [1]. This model was used to develop an hypothesis of the selective mechanism explaining the evolution of large eyes in the giant squid [1], concluding that large eyes perform best in detecting large luminous objects in the deep sea. As sperm whale (*Physeter macrocephaus*), a predator of giant squid [19-23], may appear as luminous extended targets due to mesopelagic zooplankton bioluminescence, it was inferred that predator detection was the main driver of extreme eye size evolution in *Architeuthis*.

Predictions from a theoretical model depend on the accuracy of constants to be used, a problem in particular for any model dealing with systems such as the deep sea where reliable data are difficult to obtain. Given this constraint, the robustness of the conclusions drawn from the original model was confirmed by relaxing the range of each constant value at a time [1]. However, the possibility of more than one constant being inaccurate at the same time was not considered. In order to test whether the simultaneous modification of parameters affects the results of the model, we relaxed all constants at the same time on the basis of ranges provided in the original model. We found differences from the original result that would reverse the conclusions of the paper (Figure 1b) and hence deemed it necessary to explore the parameter space of the model in more detail. In addition, we attempted to improve the accuracy of the constants in the model. We optimized parameter estimates for the visual performance model by extensive literature search, focusing on eye size, bioluminescent photon flux intensity, and mesopelagic zooplankton density. Information on in particular the two latter parameters in the deep-sea is scarce but given the available data it is appropriate to explore in how far the optimized constants may alter the original results of the optical model. Other parameter values of the original model remained unchanged.

Eye size plays a key role in the optical model as it is positively correlated with visual performance. Thus it is very important to identify the appropriate size range for which visual ranges are estimated when discussing relative advantages of prey versus predator detection. In the original model an eye diameter of 27 cm was used [1], corresponding to the largest known eye of a giant squid. However, if there is any selective advantage in visually detecting sperm whale from the far, the advantage should apply mainly to maturing individuals—a feature that only applies to very old individuals would not contribute to the fitness of the species. A more critical step is to reach sexual maturity. We therefore suggest it is necessary to estimate visual performance for a range of different pupil sizes, from the maturing individual to the giant adult one. Male *Architeuthis* spp. become sexually mature before they reach 1 m in mantle length [11,24]. The eye diameter at this body size is in the range of 8 to 9.5 cm [11] with a pupil diameter of 3–4 cm, being about one third of the eyeball measured by [1]. As mentioned above the selective advantage should mainly apply to maturing individuals, i.e., for individuals with pupil sizes of up to 4 cm, and becomes much less important for larger eyes. We thus focused on visual performance at a pupil diameter of 4 cm, but also considered the entire size range of possible pupil diameters (0–15 cm) in our calculations.

The intensity of bioluminescent light sources is also correlated with visual performance. This value describes the number of photons emitted by a bioluminescent point source in a given time interval. The rate of photon emission affects the detectability of both bioluminescent point light sources and extended luminous targets. Specifically, the distance from which these targets can be seen increases with the bioluminescent photon flux. The detectability of dark extended sources is not affected by bioluminescent photon flux. Estimates of the intensity of bioluminescence in the deeper part of the mesopelagic realm, the likely habitat of giant squid as indicated by sperm whale diving behavior [25-27] and a live observation [17], are difficult. In the original model [1] a bioluminescence photon flux value of 10^11^ quanta/s for all zooplankton was used, on the basis of data for a colony of tunicate larva [28], which may be inappropriate for the deep sea. It is known that copepods are the most abundant zooplankton in both epipelagic and mesopelagic zones [29-31] and thus it is probably reasonable to use the value for copepods (10^10^ quanta/s; [28]) as a proxy for bioluminescent photon flux. The intensity may even be less (as low as 10^9^ quanta/s) when radiolarians or dinoflagellates are the most abundant zooplankton [28], suggesting a range of bioluminescent photon flux from 10^9^ to 10^11^ quanta/s. To be conservative, we chose a bioluminescent photon flux intensity of 10^10^ quanta/s and explored the effects of lower or higher intensities.

Estimates of zooplankton density also require scrutiny and generally suffer from a lack of data for the deep mesopelagic realm. Zooplankton density strongly affects the detectability of extended luminous objects. Higher densities of plankton, i.e., lower nearest neighbor distances between individual planktonic organisms in front of a large object, increase the distance from which the target can be seen. In the original model [1] the average distance between nearest-neighbor zooplankton was set at 0.3 m in the *Architeuthis* habitat, on the basis of the estimated distance in epipelagic copepod layers at depths of 16 to 160 m [32]. However, it is known that zooplankton abundance is lower in the mesopelagic zone, especially in its deeper part [29,30,33]. For example, the nearest neighbor distance between zooplankton, assuming random spatial distribution of plankton, decreases from 0.2 m at water depths of 0–50 m to 0.65 m at 750–1000 m in Aloha (Hawaii) and from 0.14 m to 0.42 m at K2 (Kuril Islands). In meso- and bathypelagic waters off Cape Verde, the density of bioluminescent zooplankton also decreased with water depth [33]. The mean nearest neighbor distance at 500–999 m water depth was 0.42 m, and approached approximately 0.45 m at 750–999 m depth, reinforcing that the value of 0.3 m used in the original model is probably too low. Regardless, zooplankton are highly patchy in the ocean and a wide range of possible zooplankton density would be encountered. On the basis of the currently available data a reasonable estimate of the range of nearest neighbor distances of zooplankton in the giant squid habitat is approximately 0.45 to 0.65 m. For the purpose of our calculations we used the intermediate nearest neighbor distance of 0.55 m and explored the effects of smaller and larger distances.

We found no unique advantage of large eyes for detecting large luminous objects when calculating maximum viewing distances in the deep mesopelagic sea with revised constants (pupil size = 4 cm; intensity of bioluminescence = 10^10^ quanta/s; zooplankton density/nearest neighbor distance = 0.55 m) (Figure 1c, d). Point light sources can be detected over a very similar distance. In general, our calculations predict viewing distances of less than the 120 m reported previously [1]. Even at a pupil size of 15 cm the maximum viewing distance does not exceed ~80 m (Figure 1d) and that distance decreases to about 50 m for a pupil size of 4 cm. Given these low visual ranges, future studies should attempt to calculate the minimum detection distance that is required for a successful escape of giant squid from attacking sperm whale. In contrast to previous results [1] eyes with pupil sizes ranging from 2–15 cm in diameter perform about equally well in detecting point targets against black background and luminous large (2 m diameter) objects. For example, in a giant squid with a pupil diameter of 5 cm, which is probably near the maximum pupil size of a maturing individual, the predicted viewing distance for point targets and extended luminous targets with a diameter of 2 m is nearly identical at about 50 m. For eyes with pupil sizes larger than ~6-8 cm there is a small advantage for point light detection (Figure 1d). We also explored in how far varying the parameters within the ranges that likely are encountered in the habitat of giant squid, i.e., bioluminescent intensity from 10^9^ to 10^11^quanta/s and nearest neighbor distances of 0.45-0.65 m, influenced estimated visual performance. We found that an advantage for detection of large luminous objects can only be identified when maximizing bioluminescent intensity and minimizing the nearest neighbor distance of plankton. In contrast, minimizing bioluminescent intensity and maximizing nearest neighbor distances within these ranges yields an advantage for the detection of point light sources (Figure 1e, f). The results presented in Figure 1c, with conservative, intermediate parameter values seem to represent the most appropriate estimates of visual performance in the deep mesopelagic sea. All in all, it seems difficult to identify a single optical function that performs best in the mesopelagic zone.

In contrast, we emphasize the general value of large eyes in dim-light environments. The revised model calculations indicate that there are multiple optical advantages of having large eyes in the deep mesopelagic realm, i.e., 600–1000 m water depth, benefitting several visual functions related to light sensitivity. Larger eyes with larger pupils perform equally well in the detection of point-light flashes and form illumination. The viewing range for the detection of dark, large objects also increases with eye size, even though to a lesser degree (Figure 1d). For all three visual tasks, the improvement becomes smaller for larger eyes but there still is a noticeable increase in performance. Empirical evidence across vertebrates suggests that the evolution of large relative eye size is at least partially correlated with activity in dim-light environments [34-38]. Thus we predict that differences in relative eye size among squid are related to habitat (e.g., deep sea) and diel activity patterns, all of which determine the visual environment of the organism. This can be formally tested in an explicit phylogenetic framework once a well-supported species-level phylogeny and detailed life-history data become available. Such an analysis could be combined with a careful investigation of the possible influence of other predator–prey relations between cetaceans and cephalopods on eye size. On the basis of stomach contents, the diet of cetaceans includes up to 60 cephalopod species [39], providing ample resources for comparative studies.

## Conclusions

Our regression analysis suggests that the evolution of giant eyes in giant squid is largely a consequence of the evolution of giant body size. The eyes of giant squid do not seem to be unusually large—many squid, small or large, have giant eyes for their body size. We show that large eyes perform equally well in detecting point targets and form illumination in the deep sea. Whatever the cause of large eyes, they appear to have several advantages for vision in the reduced light of the mesopelagic zone.

## Competing interests

The authors have no competing interests to declare.

## Authors’ contributions

LS, RM, CEO, MDM, and PCW designed the study. LS, CEO, ARG, and JJL collected data, LS, RM, and CHM performed analyses, and LS and RM wrote the manuscript. All authors read and approved the final manuscript.

## Supplementary Material

Additional file 1 Eye size and mantle length data on squid. Click here for file

Additional file 2 **References used in Additional file 1.** Click here for file

Additional file 3 Eye size and body mass data on squid and acanthomorph fish. Click here for file

Additional file 4 Acanthomorph eye diameter, measured in-situ and enucleated. Click here for file

## References

[B1] Nilsson D-E Warrant EJ Johnsen S Hanlon R Shashar N A unique advantage for giant eyes in giant squid Curr Biol 2012 22 1 6 10.1016/j.cub.2012.02.031 22425154

[B2] Partridge JC Sensory ecology: giant eyes for giant predators Curr Biol 2012 22 R268 R270 10.1016/j.cub.2012.03.021 22537628

[B3] Warton DI Wright IJ Falster DS Westoby M Bivariate line-fitting methods for allometry Biol Rev 2006 81 259 291 10.1017/S1464793106007007 16573844

[B4] McArdle BH The structural relationship: regression in biology Can J Zool 1988 66 2329 2339 10.1139/z88-348

[B5] R Development Core Team: R A language and environment for statistical computing 2011 Vienna, Austria: R Foundation for Statistical Computing http://www.R-project.org/

[B6] Taskinen S Warton DI Robust estimation and inference for bivariate line-fitting in allometry Biom J 2011 53 4 652 672 10.1002/bimj.201000018 21681982

[B7] Warton DI Duursma RA Falster DS Taskinen S smatr 3 - an R package for estimation and inference about allometric lines Methods Ecol Evol 2012 3 257 259 10.1111/j.2041-210X.2011.00153.x

[B8] Voss GL A review of the cephalopods of the Gulf of Mexico B Mar Sci Gulf Caribb 1956 6 85 178

[B9] Sivak JG West JA Campbell MC Growth and optical development of the ocular lens of the squid (*Sepioteuthis lessoniana*) Vision Res 1994 34 2177 2187 10.1016/0042-6989(94)90100-7 7941414

[B10] Howland HC Merola S Basarab JR The allometry and scaling of the size of vertebrate eyes Vision Res 2004 44 2043 2065 10.1016/j.visres.2004.03.023 15149837

[B11] Guerra A González AF Dawe EG Rocha F Records of giant squid in the north-eastern Atlantic, and two records of male *Architeuthis* sp. off the Iberian Penninsula J Mar Biol Ass U K 2004 84 427 431 10.1017/S0025315404009397h

[B12] Andriguetto JM Jr Haimovici M Effects of fixation and preservation methods on the morphology of a loliginid squid (Cephalopoda: Myopsida) Am Malacol Bull 1988 6 213 217

[B13] Motani R Rothschild BM Wahl W Jr Large eyeballs in diving ichthyosaurs Nature 1999 402 747 10.1038/45435 10927017

[B14] Zeidberg L Allometry measurements from in situ video recordings can determine the size and swimming speeds of juvenile and adult squid *Loligo opalescens* (Cephalopoda: Myopsida) J Exp Biol 2004 207 4195 4203 10.1242/jeb.01276 15531640

[B15] Lindgren AR Pankey MS Hochberg FG Oakley TH A multi-gene phylogeny of Cephalopoda supports convergent morphological evolution in association with multiple habitat shifts in the marine environment BMC Evol Biol 2012 12 129 10.1186/1471-2148-12-129 22839506 PMC3733422

[B16] Takahashi M Okamura H Yokawa K Okazaki M Swimming behavior and migration of a swordfish recorded by an archival tag Mar Freshwater Res 2003 54 527 534 10.1071/MF01245

[B17] Kubodera T Mori K First-ever observations of a live Giant Squid in the wild Proc R Soc B 2005 272 2583 2586 10.1098/rspb.2005.3158 16321779 PMC1559985

[B18] Barel CDN Form-relations in the context of constructional morphology: the eye and suspensorium of lacustrine Cichlidae (Pisces, Teleostei): with a discussion on the implications for phylogenetic and allometric form-interactions Neth J Zool 1984 434 439 502

[B19] Clarke MR Pascoe PL Cephalopod species in the diet of a sperm whale (*Physeter catodon*) stranded at Penzance, Cornwall J Mar Biol Ass U K 1997 77 1255 1258 10.1017/S0025315400038819

[B20] Clarke MR Young R Description and analysis of cephalopod beaks from stomachs of six species of odontocete cetaceans stranded on Hawaiian shores J Mar Biol Ass U K 1998 78 623 641 10.1017/S0025315400041667

[B21] Santos MB Pierce GJ Boyle PR Reid RJ Ross HM Patterson IAP Kinze CC Tougaard S Lick R Piatkowski U Hernandez-Garcia V Stomach contents of sperm whales *Physeter macrocephalus* stranded in the North Sea 1990–1996 Mar Ecol Prog Ser 1999 183 281 294 10.3354/meps183281

[B22] Fernández R Santos MB Carrillo M Tejedor M Pierce GJ Stomach contents of cetaceans stranded in the Canary Islands 1996–2006 J Mar Biol Ass U K 2009 89 873 883 10.1017/S0025315409000290

[B23] Spitz J Cherel Y Bertin S Kiszka J Dewez A Ridoux V Prey preferences among the community of deep-diving odontocetes from the Bay of Biscay, Northeast Atlantic Deep-Sea Res I 2011 58 273 282 10.1016/j.dsr.2010.12.009

[B24] Hoving HJT Roeleveld MAC Lipinski MR Melo Y Reproductive system of the giant squid *Architeuthis* in South African waters J Zool Lond 2004 264 153 169 10.1017/S0952836904005710

[B25] Amano M Yoshioka M Sperm whale diving behavior monitored using a suction-cup-attached TDR tag Mar Ecol Prog Ser 2003 258 291 295 10.3354/meps258291

[B26] Watwood SL Miller PJO Johnson M Madsen PT Tyack PL Deep-diving foraging behaviour of sperm whales (*Physeter macrocephalus*) J Anim Ecol 2006 75 814 825 10.1111/j.1365-2656.2006.01101.x 16689963

[B27] Aoki K Amano M Mori K Kourogi A Kubodera T Miyazaki N Active hunting by deep-diving sperm whales: 3D dive profiles and maneuvers during bursts of speed Mar Ecol Prog Ser 2012 444 289 301 10.3354/meps09371

[B28] Widder EA Bioluminescence and the pelagic visual environment Mar Fresh Behav Physiol 2002 35 1 26 10.1080/10236240290025581

[B29] Böttger-Schnack R Vertical structure of small metazoan plankton, especially non-calanoid copepods. I. Deep Arabian Sea J Plank Res 1996 18 1073 1101 10.1093/plankt/18.7.1073

[B30] Steinberg DK Cope JS Wilson SE Kobari T A comparison of mesopelagic mesozooplankton community structure in the subtropical and subarctic North Pacific Ocean Deep-Sea Res II 2008 55 1615 1635 10.1016/j.dsr2.2008.04.025

[B31] Eden BR Steinberg DK Goldthwait SA McGillicuddy DJ Jr Zooplankton community structure in a cyclonic and mode-water eddy in the Sargasso Sea Deep-Sea Res I 2009 56 1757 1776 10.1016/j.dsr.2009.05.005

[B32] Widder EA Johnsen S 3D spatial point patterns of bioluminescent plankton: a map of the 'minefield' J Plank Res 2000 22 409 420 10.1093/plankt/22.3.409

[B33] Priede IG Bagley PM Way S Herring PJ Partridge JC Bioluminescence in the deep sea: free-fall lander observations in the Atlantic Ocean off Cape Verde Deep-Sea Res I 2006 53 1272 1283 10.1016/j.dsr.2006.05.004

[B34] Garamszegi LZ Møller AP Erritzøe J Coevolving avian eye size and brain size in relation to prey capture and nocturnality Proc R Soc B 2002 269 961 967 10.1098/rspb.2002.1967 12028780 PMC1690973

[B35] Thomas RJ Székely T Powell RF Cuthill IC Eye size, foraging methods and the timing of foraging in shorebirds Funct Ecol 2006 20 157 165 10.1111/j.1365-2435.2006.01073.x

[B36] Werner YL Seifan T Eye size in geckos: asymmetry, allometry, sexual dimorphism, and behavioral correlates J Morphol 2006 267 1486 1500 10.1002/jmor.10499 17117406

[B37] Schmitz L Wainwright PC Nocturnality constrains morphological and functional diversity in the eyes of reef fishes BMC Evol Biol 2011 11 338 10.1186/1471-2148-11-338 22098687 PMC3240680

[B38] Pearce E Dunbar R Latitudinal variation in light levels drives human visual system size Biol Lett 2012 8 90 93 10.1098/rsbl.2011.0570 21795263 PMC3259958

[B39] Clarke MR Cephalopods as prey. III. Cetaceans Phil Trans Roy Soc B 1996 351 1053 1065 10.1098/rstb.1996.0093

