# Supplementary material for: Allometry indicates giant eyes of giant squid are not exceptional
Source: BMC Evol Biol. 2013 Feb 18;13:45. doi: 10.1186/1471-2148-13-45 (PMC3661360; doi:10.1186/1471-2148-13-45)
Supplement: Additional file 2 — References used in Additional file 1. [file 1471-2148-13-45-S2.doc]

**Additional References**

Akyol O, Şen, H: **A new large pelagic squid record for the Northern Aegean Sea of Turkey; neon flying squid, *Ommastrephes bartrami* (LeSueur, 1821) (Cephalopoda: Ommastrephidae).** *Turk. J. Fish. Aquat. Sc.* 2004, **4**:111-113.

Arkhipkin AI, Laptikhovsky V: **Discovery of the fourth species of the enigmatic chiroteuthid squid *Asperoteuthis* (Cephalopoda: Oegopsida) and extension of the range of the genus to the South Atlantic.** *J. Mollus. Stud.* 2008, **74**:203-207.

Glaubrecht M, Salcedo-Vargas MA: **The Humboldt squid *Dosidicus gigas* (Orbigny, 1835): history of the Berlin specimen, with a reappraisal of other (bathy-)pelagic “gigantic” cephalopods (Mollusca, Ommastrephidae, Architeuthidae).** *Mitt. Mus. Nat.kd. Berl., Zool. Reihe* 2004, **80**:53-69.

González M, Fernández-Casado M, Rodríguez MdP, Segura A, Martín JJ: **First record of giant squid *Architeuthis* sp. (Architeuthidae) in the Mediterranean Sea.** *J. Mar. Biol. Ass. U.K.* 2000, **80**:745-746.

Guerra A, González AF, Dawe EG, Rocha F: **Records of giant squid in the north-eastern Atlantic, and two records of male *Architeuthis* sp. off the Iberian Penninsula.**  *J. Mar. Biol. Ass. U. K.* 2004, **84**:427-431.

Harman RF, Seki MP: ***Iridoteuthis iris* (Cephalopoda: Sepiolidae): new records from the Central North Pacific and first description of the adults.** *Pac. Sci.* 1990, **44**:171-179.

Ho C-W, Lu C-C:. **Two new species of *Sepia* (*Doratosepion*) (Cephalopoda: Sepiidae) from Taiwan, based on morphological and molecular data.** *Phuket mar. biol. Cent. Res. Bull.* 2005, **66**:51-69.

Hoving HJT, Roeleveld MAC, Lipinski MR, Melo Y: **Reproductive system of the giant squid *Architeuthis* in South African waters.**  *J. Zool. Lond.* 2004,**264**:153-169.

Hylleberg J, Nateewathma A: **Morphology, internal anatomy, and biometrics of the cephalopod *Idiosepius biserialis* Voss, 1962. A new record for the Andaman Sea.** *Phuket mar. biol. Cent. Res. Bull.* 1991, **56**:1-9.

Judkins H, Ingrao DA, Roper CFE: **First records of *Asperoteuthis acanthoderma* (Lu, 1977) (Cephalopoda: Oegopsida: Chiroteuthidae), from the North Atlantic Ocean, Straits of Florida.** *Proc. Biol. Soc. Wash.* 2009, **122**:162-170.

Kasim MK, Marichamy R, Rajapackiam S, Balasubramanian TS: **Rare squid, *Thysanoteuthis rhombus* Troschel from the Gulf of Manna, India.** *J. Mar. Biol. Ass. India* 1998**39**, 182-184.

McSweeny ES: **Description of the juvenile form of the Antarctic squid *Mesonychoteuthis hamiltoni* Robson.** *Malacologia* 1970, **10**:323-332.

Marčić Z, Ćaleta M, Buj I, Mrakovčić M, Mustafić P, Zanella D, Dulčić J:. **First record of *Thysanoteuthis rhombus* (Cephalopoda:Thysanoteuthidae) in the Adriatic Sea.** JMBA2 – Biodiversity Records; 2008.

Mensch R: **A systematic review of the squid genus *Chiroteuthis* (Mollusca: Cephalopoda) in New Zealand waters.** *Masters Thesis*. Auckland University of Technology; 2010.

Neethiselvan N: **A new species of cuttlefish *Sepia ramani* sp. nov. (Class: Cephalopoda) from Tuticorin Bay, southeast coast of India.** *Indian J. Mar. Sci.* 2001, **30**:81-86.

Neethiselvan N, Venkataramani VK: ***Sepia prabahari* sp. nov. (Mollusca/Cephalopoda), a new species of Acanthosepion species complex from Tuticorin bay, southeast coast of India.** *Indian J. Mar. Sci.* 2002, **31**:45-51.

Reid A: **Taxonomic review of the Australian Rossiinae (Cephalopoda: Sepiolidae), with a description of a new species, *Neorossia leptodons*, and redescription of *N. caroli* (Joubin, 1902).** *B. Mar. Sci.* 1991, **49**:748-831.

Reid A: ***Sepioloidea magna* sp. nov.: a new bobtail squid (Cephalopoda: Sepiadariidae) from northern Australia.** *Research Online, University of Wollongong* **2009**:103-109. (http://ro.uow.edu.au/scipapers/296)

Reid A, Lu C-C: **A new cuttlefish, *Sepia filibranchia* n. sp., from the South China Sea with a redescription of *Sepia mestus* Gray, 1849 (Cephalopoda: Sepiidae) from eastern Australia.** *Zootaxa* 2005, **911**:1-22.

Rodhouse PG, Lu CC: ***Chiroteuthis veranyi* from the Atlantic sector of the Southern Ocean (Cephalopoda: Chiroteuthidae).** *S. Afr. J. Mar. Sci.* 1998, **20**:311-322.

Sreenivasan PV, Sarvesan R: **On the cephalopods collected during the exploratory survey by FORV Sagar Sampada in the Andaman-Nicobar Seas.** *Proc. First Workshop Scient. Resul. FORV Sagar Sampada, 5-7 June 1989*;1990:409-413.

Tsuchiya K, Mori K: **A note on an early juvenile specimen of *Architeuthis* sp. collected from the south off central Honshu, Japan (Cephalopoda: Architeuthidae).** *VENUS (Jap. Jour. Malac.)* 1998, **57**:225-230.

Vecchione M, Young RE: **The squid family Magnapinnidae (Mollusca: Cephalopoda) in the Atlantic Ocean, with a description of a new species.** *P. Biol. Soc. Wash.* 2006, **119**:365-372.

Vecchione M, Young RE: **The Magnapinnidae, a newly discovered family of oceanic squid (Cephalopoda: Oegopsida).** *S. Afr. J. Mar. Sci.* 1998, **20**:429-437.

Voss GL: Bermudan cephalopods. *Fieldiana Zool.* 1960, **39**:419-446.

Young RE: **The systematics and areal distribution of pelagic cephalopods from the seas off southern California.** *Sm. C. Zool.* 1972, **97**:1-159.

Young RE, Vecchione M, Roper CFE: **A new genus and three new species of decapodiform cephalopods (Mollusca: Cephalopoda).** *Rev. Fish Biol. Fisheries* 2007, **17**:353-365.
